# Expression of TMEM16A in Colorectal Cancer and Its Correlation With Clinical and Pathological Parameters

**DOI:** 10.3389/fonc.2021.652262

**Published:** 2021-03-19

**Authors:** Hongxia Li, Qiwei Yang, Sibo Huo, Zhenwu Du, Fei Wu, Haiyue Zhao, Shifan Chen, Longfei Yang, Zhiming Ma, Yujie Sui

**Affiliations:** ^1^Department of Dermatology, First Hospital of Jilin University, Changchun, China; ^2^Key Laboratory for Molecular and Chemical Genetics of Critical Human Diseases of Jilin Province, Second Hospital of Jilin University, Changchun, China; ^3^Department of Gastrointestinal Nutrition and Hernia Surgery, Second Hospital of Jilin University, Changchun, China; ^4^Department of General Surgery, Qian Wei Hospital of Jilin Province, Changchun, China; ^5^Department of Orthopedics, Second Hospital of Jilin University, Changchun, China; ^6^Department of Gynecology and Obstetrics, Second Hospital of Jilin University, Changchun, China; ^7^Center of Reproductive Medicine and Center of Prenatal Diagnosis, First Hospital of Jilin University, Changchun, China; ^8^Department of Pathology, Second Hospital of Jilin University, Changchun, China

**Keywords:** TMEM16A, calcium activated chloride channel, colorectal cancer, lymph node metastasis, immunohistochemistry

## Abstract

TMEM16A is a recently identified calcium-activated chloride channel (CaCC) and its overexpression contributes to tumorigenesis and progression in several human malignancies. However, little is known about expression of TMEM16A and its clinical significance in colorectal cancer (CRC). TMEM16A mRNA expression was determined by quantitative real time-PCR (qRT-PCR) in 67 CRC tissues and 24 para-carcinoma tissues. TMEM16A protein expression was performed by immunohistochemistry in 80 CRC tissues. The correlation between TMEM16A expression and clinicopathological parameters, and known genes and proteins involved in CRC was analyzed. The results showed that TMEM16A mRNA expression was frequently detected in 51 CRC tissues (76%), whereas TMEM16A protein expression was determined at a relatively lower frequency (26%). TMEM16A mRNA expression in tumor tissues was higher than its expression in normal para-carcinoma tissues (*P* < 0.05). TMEM16A mRNA expression was significantly correlated with TNM stage (*p* = 0.039) and status of lymph node metastasis (*p* = 0.047). In addition, there was a strong positive correlation between TMEM16A mRNA expression and MSH2 protein. More importantly, TMEM16A protein expression was positively associated with KRAS mutation, and negatively correlated with mutant p53 protein. Logistic regression analysis demonstrated that TMEM16A mRNA expression was an important independent predictive factor of lymph node metastasis (OR = 16.38, CI: 1.91–140.27, *p* = 0.01). TMEM16A mRNA and protein expression was not significantly related with patient survival. Our findings provide original evidence demonstrating TMEM16A mRNA expression can be a novel predictive marker of lymph node metastasis and TMEM16A protein expression may be an important regulator of tumor proliferation and metastasis in CRC.

## Introduction

Colorectal carcinomas (CRC) is one of the most prevalent malignancy worldwide, and the third most deadly cancer (1). Despite the significant advancement in diagnosis and treatment of colorectal cancer over the past decade, the survival rate of advanced colorectal cancer remains poor owing to tumor recurrence, distant metastasis, and lack of diagnosis markers. Therefore, there is an urgent need for new prognostic and diagnostic markers and therapeutic targets, and a better understanding of molecular mechanisms underlying tumor development and progression.

TMEM16A, also known as ANO1, DOG1, ORAOV2, or TAOS2, was identified as a novel molecular component of calcium-activated chloride channel (CaCC) in 2008 (2–4). TMEM16A regulates many cellular functions, including epithelial secretion, cardiac, and neuronal excitation, smooth muscle contraction (5–8). Before the discovery of TMEM16A as a CaCC, TMEM16A has been described as a biomarker for gastrointestinal stromal tumor (GIST) (9, 10). Recently, growing evidences have shown that TMEM16A is overexpressed or amplified in many tumors, such as head and neck squamous cancer cells (HNSCC), esophageal squamous cell cancer (ESCC), and breast cancer (11–17), and overexpression of TMEM16A plays an important role in the development and progression of tumors (18–22). Duvvuri et al. found that TMEM16A overexpression correlated with decreased overall survival in patients with HNSCC (19). Ayoub et al. found ANO1 amplification and expression in human papilloma virus (HPV)-negative HNSCC accompanied with a high propensity for future distant metastasis (20). Shi et al. reported that TMEM16A mRNA expression and protein overexpression was associated with lymph node metastasis and advanced clinical stage in ESCC patients (21). Bae et al. found that TMEM16A expression is associated with shorter survival and progression of breast cancer (22). Thus, TMEM16A was thought as a new promising prognostic and diagnostic marker and potential therapeutic target for the treatment of some types of cancers. However, current available information is very limited regarding biological function and clinical significance of TMEM16A expression in CRC.

In our previous study, we found that TMEM16A expression in colorectal cancer SW620, HCT116, and LS174T cells, not in SW480 and HCT8 cells, and inhibition of TMEM16A expression decreased the growth, migration, and invasion ability of SW620 cells (23). These interesting findings prompted us to investigate clinical relevance of TMEM16A expression in CRC tissues. In the present study, we detected TMEM16A mRNA expression and protein expression in clinical CRC samples. Then, we analyzed the correlations of TMEM16A expression with clinicopathological parameters and patient prognosis. Finally, we investigated the relation between TMEM16A and tumor proliferation and metastasis related molecules, including KRAS/NRAS/BRAF mutations status, and expression of known proteins involved in CRC (Braf, CDX2, EGFR, p53, Ki67, CD34, MLH1, MSH2, MSH6, and PMS2).

## Materials and Methods

### Patients and Tissue Samples

A total of 171 CRC tissue specimens, including 80 CRC samples for IHC, 67 CRC samples, and 24 normal colorectal samples for real-time PCR, were retrospectively collected in this study. Patients underwent resection of their tumors and were pathologically confirmed as CRC at the Second Hospital of Jilin University between November 2016 and June 2018. Informed consent were obtained from all these patients. No patients received adjuvant treatment prior to surgery. Clinical information was obtained by reviewing medical records and pathologic reports. An overview of all clinicopathological data of these patients given in Table 1. All involved experiments were performed in accordance with relevant guidelines and regulations of the Ethics Committee of the Second Hospital of Jilin University.

**Table 1:** Clinicopathological characteristics of the CRC patients.

	**TMEM16A mRNA expression (qPCR)**			**TMEM16A protein expression (IHC)**		
**Characteristics**	**Colon cancer (*n* = 21)**	**Rectal cancer (*n* = 46)**	**Total (*n* = 67)**	**Colon cancer (*n* = 32)**	**Rectal cancer (*n* = 48)**	**Total (*n* = 80)**
	***n* (%)**	***n* (%)**	***n* (%)**	***n* (%)**	***n* (%)**	***n* (%)**
**Age at surgery (years)**						
Mean ± SD	62.14 ± 11.13	61.95 ± 10.99	61.85 ± 10.87	62.31 ± 10.14	61.94 ± 10.35	61.86 ± 10.27
Median	63 (range, 44–79)	63 (range, 37–83)	63 (range, 37–83)	63 (range, 44–79)	63.5 (range, 37–83)	63 (range, 37–83)
<60	9 (42.9)	20 (43.5)	29 (43.3)	10 (31.2)	23 (47.9)	33 (41.3)
≥60	12 (57.1)	26 (56.5)	38 (56.7)	22 (68.8)	25 (52.1)	47 (58.7)
**Gender**						
Male	14 (66.7)	32 (69.6)	46 (68.7)	21 (65.6)	34 (70.8)	55 (68.8)
Female	7 (33.3)	14 (30.4)	21 (31.3)	11 (34.4)	14 (29.2)	25 (31.2)
**Histological grade**						
I	0 (0.0)	7 (15.2)	7 (10.4)	1 (3.1)	6 (12.5)	7 (8.8)
II	13 (61.9)	12 (26.1)	25 (37.3)	20 (62.5)	16 (33.3)	36 (45)
III	7 (33.3)	27 (58.7)	34 (50.7)	10 (31.3)	26 (54.2)	36 (45)
IV	1 (4.8)	0 (0.0)	1 (1.5)	1 (3.1)	0 (0.0)	1 (1.2)
**Differentiation grade**						
Well	0 (0.0)	1 (2.2)	1 (1.5)	0 (0.0)	2 (4.2)	2 (2.5)
Medium	13 (61.9)	32 (69.6)	45 (67.2)	19 (59.4)	32 (66.7)	51 (63.8)
Poor	8 (38.1)	13 (28.2)	21 (31.3)	13 (40.6)	14 (29.1)	27 (33.7)
**Lymphnode metastasis**						
N0	13 (61.9)	19 (41.3)	32 (47.8)	21 (65.6)	22 (45.8)	43 (53.7)
N1	5 (23.8)	11 (23.9)	16 (23.9)	8 (25.0)	11 (22.9)	19 (23.8)
N2	3 (14.3)	16 (34.8)	19 (28.3)	3 (9.4)	15 (31.3)	18 (22.5)

### Immunohistochemistry Staining

Eighty CRC tissue specimens were fixed in 4% buffered formaldehyde and embedded in paraffin. The tissues were cut into 2-μM sections and dewaxed, hydrated, and antigen retrievaled by PT link (Dako, Agilent Technologies, USA). Primary antibodies, secondary antibodies and DAB staining was done at room temperature on an automatic station workstation (Dako, Agilent Technologies, USA). Primary antibodies for TMEM16A, Braf (V600E), EGFR, MSH2, MLH1, Tp53, Ki67, CDX2, and MSH6 were purchased from Zsbio company (Beijing, China). Finally, all sections counterstained with hematoxylin for 1 min.

Tissue specimens were observed with a light microscope (Olympus BX51) by 2 pathologists without prior knowledge of patient data. The IHC staining results were assigned a mean score based on both the intensity of staining and the percentage of positive cells. The IHC intensity was scored as follows: 0, 1, 2, and 3 points indicated no staining, minimal staining (light yellow), moderate staining (yellow brown), and strong staining (brown), respectively. The percentage of positive cells was determined using a previously reported method, and the cells were divided into four groups: 0, 1, 2, 3, and 4 points indicated < 5% positive cells, < 5–25% positive cells, 26–50% positive cells, 51–75% and 76–100% positive cells. The IHC was scored using a composite scoring system: scores were calculated by multiplying the intensity with the percentage of positive cells having this intensity. 0, 1–4, 5–8, 9–12 points was considered as negative, weak/mild, moderate/medium, and strong. For statistical analysis, samples with a score >0 were classified as IHC positive.

### Quantitative Reverse-Transcription Polymerase Chain Reaction

Total RNA was extracted from 67 CRC tissues and 24 normal colorectal tissues using Trizol reagent (Invitrogen Life Technologies). Two micrograms total RNA were subjected to reverse transcription using cDNA Synthesis Kit (Genecopeia, USA). Real-time PCR was performed with SYBR Green PCR master mix (Genecopeia, USA) and ABI 7500 Fast Dx (Applied Biosystems Co. Ltd., USA). All experiments were carried out in triplicate, and the results were normalized to the expression of glyceraldehyde-3-phosphate dehydrogenase (GAPDH). The following primers were used: TMEM16A, sense primer, 5′-GATCCCATCCAGCCCAAAGTG-3′; antisense primer, 5′-CGGGTTTTGCTGTC GAAAAAGGA-3′; GAPDH, sense primer, 5′-CGGACCAATACGACCAAATCCG-3′; antisense primer, 5′-AGCCACATCGCTCAGACACC-3′. Dissociation curve analysis of all PCR products showed a single sharp peak and the size of each amplified product was confirmed by ethidium bromide-stained agarose gel electrophoresis. TMEM16A was calculated using 2^−ΔCt^ method. The ΔCt represents the average Ct for the target gene (TMEM16A) minus the average Ct for the reference gene (GAPDH). Values higher than 0.001 were considered positive for mRNA expression. The mRNA expression was further classified as low expression (values between 0.001 and 0.01), medium expression (values between 0.01 and 0.1) and high expression (values above 0.1).

### DNA Extraction and Mutation Detection

Genomic DNA was extracted from surgical colorectal tumor tissue. The TIANamp Genomic DNA Kit (Tiangen Biotech, Beijing, China) were used following the manufacturer's protocol.

For each CRC sample, mutations of KRAS exons 2 (codon 12 and 13), mutations of NRAS exons 2 (codon 12 and 13), exons 3 (codon 59 and 61), exons 4 (codon 117 and 146), and mutations of BRAF exons 15 (codon 600) were detected according to the manufacturer's instructions. Human Gene Mutation Detection Kit of KRAS, NRAS, and BRAF were purchased from YZY Medical Science & Technology Co., Ltd. (Wuhan, China). Real-time quantitative polymerase chain reaction was performed by ABI 7500 Fast Dx (Applied Biosystems Co. Ltd., US).

### Statistical Analysis

The SPSS version 21 (SPSS Inc., USA) software was used for the statistical analysis. All data are presented as mean ± SEM unless stated otherwise. A paired *t* test was used to test for differences in TMEM16A expression between matched CRC tissues and normal colorectal tissues. Correlation between mRNA expression and protein expression was explored by a Spearman's correlation. Categorical variables were compared by ANOVA or Fisher's exact test. Quantitative and ordered variables were compared by the Mann-Whitney test. The Kaplan-Meier (KM) method were used to evaluate the time to diagnosis of overall survival. *P* < 0.05 were considered to be statistically significant.

## Results

### TMEM16A Protein Expression and mRNA Expression in CRC

**Figure 1:**
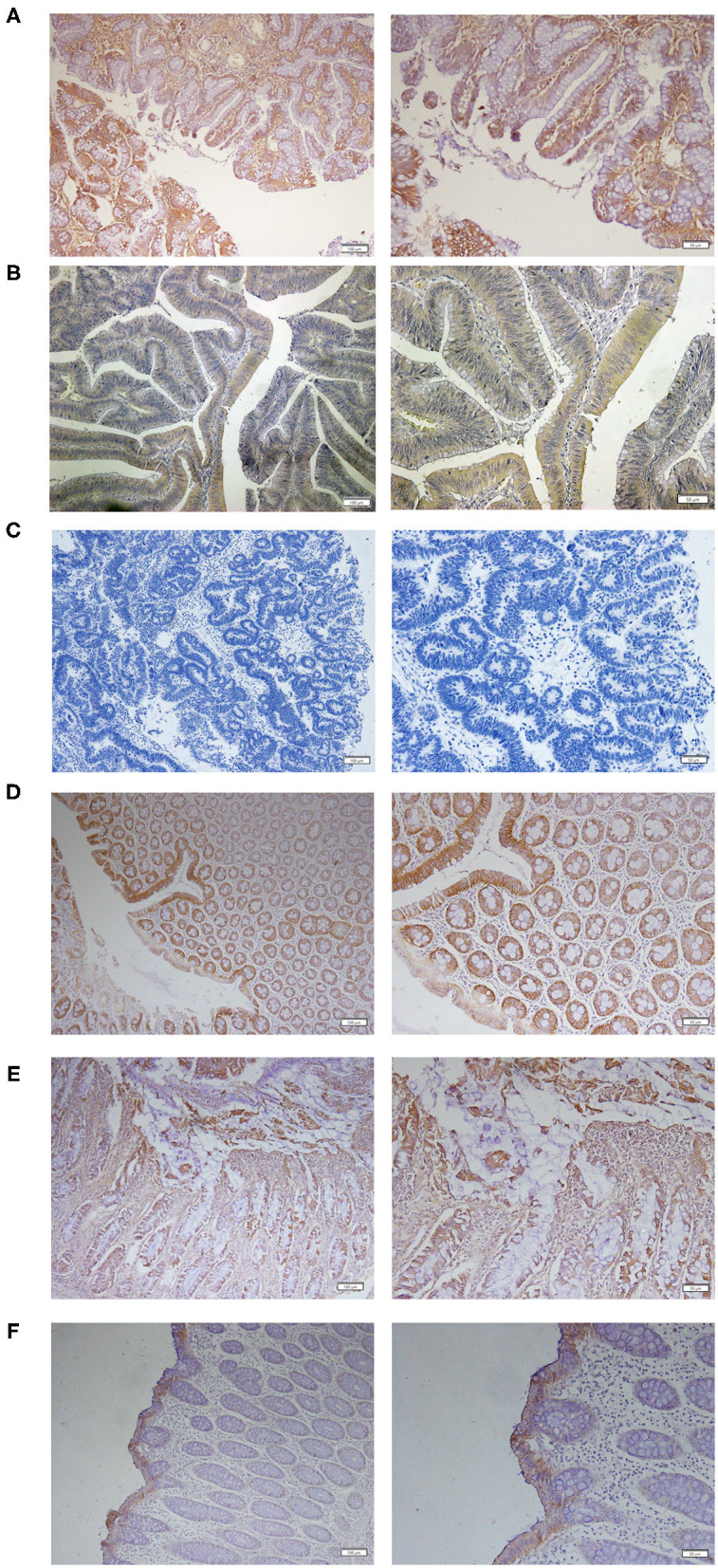
Expression of TMEM16A in human colorectal cancer. **(A)** Representative IHC images of medium expression of TMEM16A. **(B)** Representative IHC images of low expression of TMEM16A. **(C)** Representative images of negative staining for TMEM16A. **(D)** Representative images of IHC staining showing TMEM16A expression in glands and mucosa of colorectal cancer tissues. **(E)** Expression of TMEM16A in glands and connective tissue of colorectal cancer. **(F)** Expression of TMEM16A on the mucosal surface of colorectal carcinoma.

Immunohistochemical analysis of TMEM16A protein expression was carried out on tumor tissues from 80 primary CRC patients. The results revealed that 21 (26%) of 80 colorectal cancer tissues exhibited positive TMEM16A expression including 16 (20%) cases with low expression and 5 (6%) cases with medium expression. High expression was not found in this study. Representative images of TMEM16A immunostaining are shown in Figure 1. The staining observed with TMEM16A antibody appeared predominately localized to the membrane and plasma. Additionally, TMEM16A was mainly expressed in the glands of colorectal cancer, except 2 cases on the surface of mucosa and 2 cases in the glands and submucosa.

**Figure 2:**
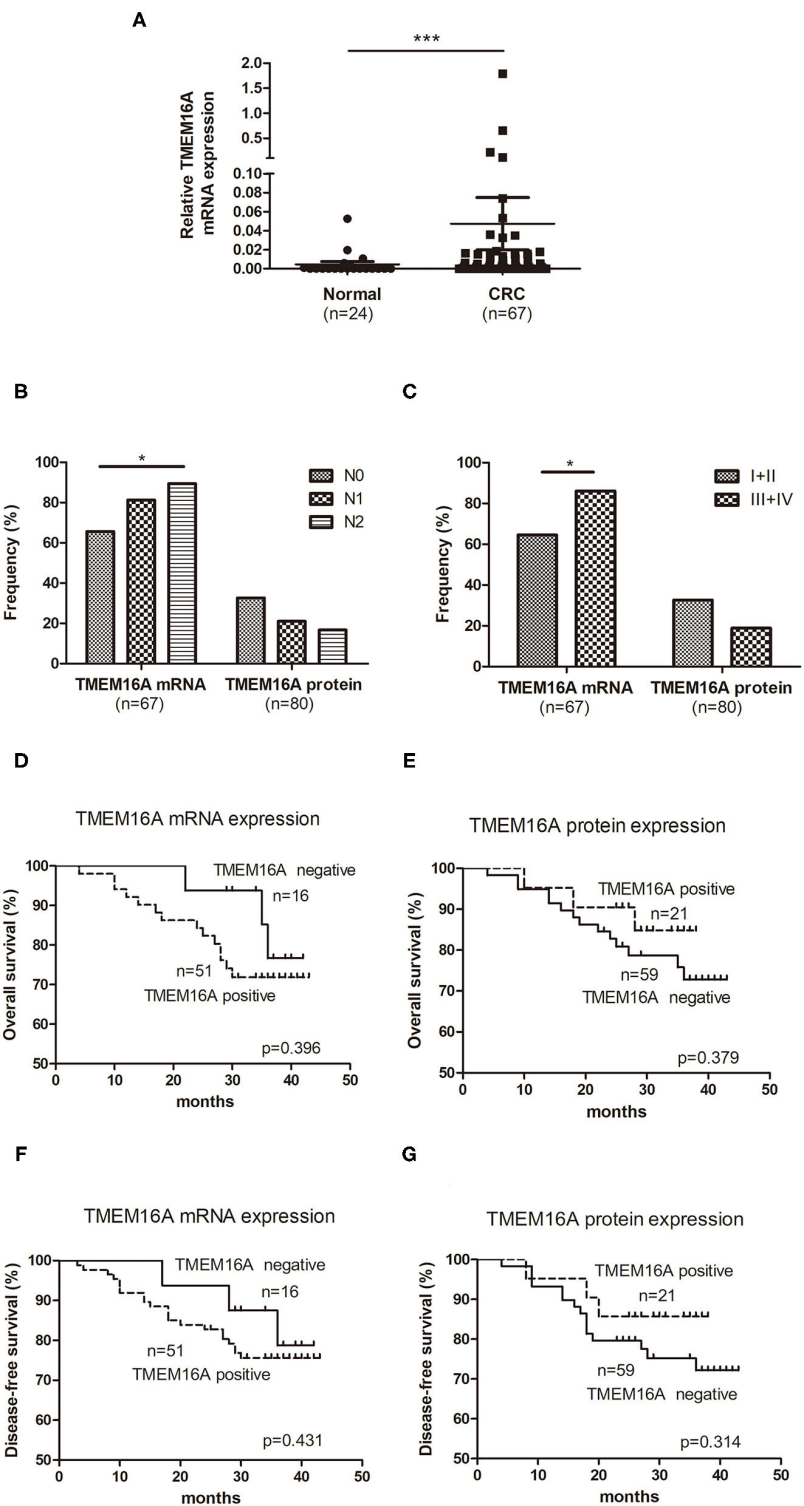
Expression of TMEM16A in colorectal carcinoma and its correlation with overall survival. **(A)** Quantitative RT-PCR results showed that the expression of TMEM16A in colorectal cancer was significantly higher than that in normal colorectal tissue (***P < 0.0001). **(B)** Positive rates of TMEM16A protein expression and mRNA expression in different lymph node status of colorectal carcinoma progression (*P < 0.05). **(C)** Positive rates of TMEM16A protein expression and mRNA expression in different stages of colorectal carcinoma progression (*P < 0.05). **(D)** Kaplan-Meier survival curve analysis showed that CRC patients with TMEM16A mRNA expression tended to a worse overall survival. **(E)** Kaplan-Meier survival curve analysis about effect of TMEM16A protein expression on patient verall survival. **(F)** The effect of TMEM16A mRNA expression on disease-free survival was analyzed by Kaplan Meier survival curve. **(G)** The effect of TMEM16A protein expression on disease-free survival was analyzed by Kaplan Meier survival curve.

TMEM16A mRNA expression was examined in 67 CRC tissue specimens and 24 normal colorectal tissue specimens by real-time PCR. The results demonstrated that TMEM16A mRNA expression was detected in 51 (76%) of 67 CRC tissue samples including 27 (40%) with low expression, 20 (30%) medium expression, and 4 (6%) high expression. Compared to normal colorectal tissues, TMEM16A expression was significantly increased in tumor tissues (Figure 2A, *P* < 0.0001). Analysis of TMEM16A mRNA expression and protein expression in CRCs is shown in Table 2. Although there was a significant positive correlation between TMEM16A mRNA expression and protein expression (*p* = 0.019), there was a weak correlation between two parameters (Spearman's = 0.337). Fifteen of 18 cases (83%) showing TMEM16A-positive expression harbored TMEM16A mRNA expression. However, more than half of the cases (19/34) with TMEM16A mRNA expression did not lead to TMEM16A protein expression. Furthermore, the level of mRNA expression is not always positively correlated with the level of protein expression (Supplementary Figure 1).

Table 2Analysis of TMEM16A mRNA and protein expression in CRCs.

**Table:** 

	**TMEM16A mRNA expression**		**TMEM16A protein expression**	
	***n***	**Percentage**	***n***	**Percentage**
**(A)**				
Negative	16	23.9	59	73.8
Low	27	40.3	16	20.0
Medium	20	29.8	5	6.2
High	4	6.0	0	0.0
Total	67	100.0	80	100.0

**Table:** 

	**TMEM16A protein expression**				
		**Evaluable (*****n*****)**	**Negative**	**Positive**	***P***
**(B)**					
TMEM16A mRNA expression	Negative	14	11 (37%)	3 (17%)	0.196
	Positive	34	19 (63%)	15 (83%)	

### TMEM16A Expression and Clinicopathological Parameters in CRCs

To explore the potential function of TMEM16A expression in CRC, we further examined the associations of TMEM16A mRNA expression and protein expression with clinicopathological parameters. The results were shown in Table 3 and Supplementary Figure 2. TMEM16A mRNA expression was significantly correlated to TNM stage (*p* = 0.039) and status of lymph node metastasis (*p* = 0.047). However, there was no significant associations between TMEM16A mRNA expression and other clinicopathological parameters, including gender, age at surgery, tumor location, histological type, and differentiation. The correlation between TMEM16A protein and any of the clinicopathological parameters was not statistically significant (*P* > 0.05).

**Table 3:** Associations between TMEM16A expression and clinical features in CRCs.

	**TMEM16A mRNA expression (qPCR)**				**TMEM16A protein expression (IHC)**			
	***n***	**Negative**	**Positive**	***P***	***n***	**Negative**	**Positive**	***P***
**Gender**								
Male	46	11	35	0.993^a^	55	40	15	0.758^a^
Female	21	5	16		25	19	6	
**Age at surgery (year)**								
<60	29	6	29	0.593^a^	33	27	6	0.169^a^
≥60	38	10	38		47	32	15	
**Tumor location**								
Colon	21	6	15	0.543^a^	32	20	12	0.062^a^
Rectal	46	10	36		48	39	9	
**Histological type**								
Adenocarcinoma	58	13	45	0.381^b^	69	50	19	0.655^b^
Mucinous adenocarcinoma	7	3	4		9	7	2	
Signet-ring cell carcinoma	2	0	2		2	2	0	
**Differentiation**								
Well	1	1	0	0.746^c^	2	1	1	0.764^c^
Moderate	45	10	35		51	39	12	
Poor	21	5	16		27	19	8	
**TNM stage**								
I+II	31	11	20	**0.039**^**a**^	43	29	14	0.167^a^
III+IV	36	5	31		37	30	7	
**T**								
T1	0	0	0	0.623^c^	1	1	0	0.664^c^
T2	7	2	5		6	5	1	
T3	54	13	41		65	47	18	
T4	6	1	5		8	6	2	
**N**								
N0	32	11	21	**0.047**^***c***^	43	29	14	0.161 ^c^
N1	16	3	13		19	15	4	
N2	19	2	17		18	15	3	

a *Chi-square test*.

b *Fisher's exact test*.

c *Mann-Whitney test*.

*Bold values means P < 0.05, which indicate the statistically significantly difference*.

We further investigated the positive rate of TMEM16A mRNA and protein expression in the process of tumor development. As Figure 2B shown, with the development of lymph node metastasis (N0, N1, N2), the positive rate of TMEM16A mRNA expression increased significantly, but the positive rate of TMEM16A protein expression did not change significantly. Moreover, the positive rate of TMEM16A mRNA expression in stage III and IV with lymph node metastasis was significantly higher than that in stage I and II without lymph node metastasis (Figure 2C) which further proved that TMEM16A mRNA expression was related to lymph node metastasis. There was no significant difference between the positive rate of TMEM16A protein expression in stage III and IV and that in stage I and II.

### Role of TMEM16A for Survival in Patients With CRC

Next, we investigated the association between TMEM16A and clinical outcome of patients with CRC. Kaplan Meier survival analysis showed no significant correlation between TMEM16A mRNA or protein expression and overall survival or disease-free survival (Figures 2D–G). Interestingly, we found that there may be a trend toward decreased survival in patients with TMEM16A mRNA expression (HR = 1.59, CI = 0.54–4.68, *p* = 0.396), whereas patients carrying lesions with TMEM16A protein expression positive seemed to have improved overall survival (HR = 0.67, CI = 0.21–1.81, *p* = 0.379). Consistently, similar results were also demonstrated in disease-free survival of patients.

### TMEM16A Expression and IHC Characteristics

Immunohistochemical markers play an important role in tumorigenesis, pathological classification, differential diagnosis of benign and malignant tumors, and prognosis evaluation of patients. To investigate the potential effect of TMEM16A on the progression of colorectal cancer, we examined the expression of the most commonly used immunohistochemical markers in the clinical diagnosis of colorectal cancer, including BRAF (V600E), CDX2, EGFR, p53, Ki67, CD34, PMS2, MLH1, MSH6, and MSH2. We further analyzed the relationship between the expression of TMEM16A and these immunohistochemical markers. The results showed that MSH2 were significantly correlated with TMEM16A mRNA expression (*p* = 0.047). Additionally, there was a strong correlation between the expression of mutant p53 protein and TMEM16A protein (*p* = 0.025). No significant associations between TMEM16A and other immunohistochemical markers were observed in our study (Table 4).

**Table 4:** TMEM16A mRNA and protein expression in relation to immunohistochemistry characteristics in CRCs.

		**TMEM16A mRNA expression (qPCR)**				**TMEM16A protein expression (IHC)**			
		***n***	**Positive**	**Negative**	***P***	***n***	**Positive**	**Negative**	***P***
BRAF(V600E)	Positive	3	2	1	0.565^a^	2	1	1	0.474^a^
	Negative	64	49	15		75	20	55	
	Missing	/	/	/		3	0	3	
CDX2	Positive	65	49	16	1.000^a^	76	21	55	0.561^a^
	Partially positive	2	2	0		3	0	3	
	Missing	/	/	/		1	0	1	
EGFR	Positive	31	21	10	0.134^b^	34	10	24	0.928^b^
	Weakly positive	14	11	3		22	4	18	
	Negative	21	18	3		19	6	13	
	Missing	1	1	0		5	1	4	
Mutant p53	Positive rate≥90%	23	17	6	0.353^b^	25	4	21	**0.025**^***b***^
	Positive rate 50–90%	10	8	2		9	1	8	
	Positive rate <50%	9	4	5		17	5	12	
	Negative	25	22	3		26	11	15	
	Missing	/	/	/		3	0	3	
Ki67	Positive rate≥90%	28	22	6	0.907^b^	34	6	28	0.296^b^
	Positive rate 80–90%	18	13	5		18	7	11	
	Positive rate 70–80%	14	10	4		14	4	10	
	Positive rate 60–70%	5	4	1		8	4	4	
	Positive rate <60%	2	2	0		5	0	5	
	Missing	/	/	/		1	0	1	
CD34	Positive	12	8	4	0.210^b^	10	3	7	0.396^b^
	Vessel positive	14	10	4		12	2	10	
	Negative	25	21	4		30	11	19	
	Missing	16	12	4		28	5	23	
MLH1	Positive	58	45	13	0.502^b^	68	19	49	0.758^b^
	Partially positive	8	5	3		7	1	6	
	Negative	1	1	0		2	1	1	
	Missing	/	/	/		3	0	3	
MSH2	Positive	62	49	13	**0.047**^**b**^	75	20	55	0.468^b^
	Partially positive	4	2	2		2	1	1	
	Negative	1	0	1		0	0	0	
	Missing	/	/	/		3	0	3	
MSH6	Positive	57	44	13	0.593^b^	68	19	49	0.758^b^
	Partially positive	8	6	2		7	1	6	
	Negative	2	1	1		2	1	1	
	Missing	/	/	/		3	0	3	
PMS2	Positive	66	50	16	1.000^a^	75	20	55	0.474^a^
	Negative	1	1	0		2	1	1	
	Missing	/	/	/		3	0	3	

a *Chi-square test or Fisher's exact test if appropriate*.

b *Mann-Whitney test*.

*Bold values means P < 0.05, which indicate the statistically significantly difference*.

### TMEM16A Expression and Mutation Status of KRAS, NRAS and BRAF

Our previous study showed that TMEM16A siRNA led to decreased *in vitro* proliferation of human colorectal cancer cells SW620 by inhibiting the expression of ERK1/2 (23). It is well-known that RAS-RAF-MAPK signaling pathway can activate ERK1/2. Mutation in KRAS, NRAS, and BRAF has been demonstrated to be involved in initiation and progression of colorectal carcinoma (24). Therefore, we wanted to confirm whether expression of TMEM16A correlates with mutation status of KRAS/NRAS/BRAF. We examined and analyzed mutation status of KRAS/NRAS/ BRAF in colorectal cancer tissues. As expected, there was strongly correlation between TMEM16A protein expression and KRAS mutation (*p* = 0.017). 13 of 32 (40.6%) KRAS mutant type CRC were positive for TMEM16A. In contrast, only 8 of 58 (16.7%) KRAS wild type CRC were positive for TMEM16A. No statistically significant association of TMEM16A protein expression with mutations in NRAS and BRAF was observed. In addition, the correlation between TMEM16A mRNA expression and mutation status of KRAS/NRAS/BRAF has not been found (Table 5).

**Table 5:** TMEM16A mRNA and protein expression according to KRAS/NRAS/BRAF mutation status in CRCs.

		**TMEM16A mRNA expression (qPCR)**				**TMEM16A protein expression (IHC)**			
		***n***	**Negative**	**Positive**	***P***	***n***	**Negative**	**Positive**	***P***
KRAS (codon 12/13)	MT	29	6	23	0.593^a^	32	19	13	**0.017**^**a**^
	WT	38	10	28		48	40	8	
NRAS (codon12/13/59/61/117/46)	MT	1	0	1	1.000^b^	3	2	1	1.000^b^
	WT	66	16	50		77	57	20	
BRAF (condon 600)	MT	1	1	0	1.000^b^	0	0	0	1.000^b^
	WT	66	16	50		80	59	21	

*MT means mutant type. WT means wild type*.

a *Chi-square test*.

b *Fisher's exact test*.

*Bold values means P < 0.05, which indicate the statistically significantly difference*.

### Univariate and Multivariate Logistic Regression Analysis Between TMEM16A and Lymph Node Metastasis

In order to further explore the correlation between TMEM16A and lymph node metastasis in CRC, univariate and multivariate logistic regression analysis were performed. In univariate logistic regression analysis, factors with possible impact to nodal disease were incorporated into the model, including clinical pathological parameters such as gender, age, location, tumor size, histological type, differentiation, EGFR, CD34, Ki67, mutant p53, MLH1,MSH2, KRAS, along with TMEM16A mRNA expression and protein expression. In multivariate logistic regression analysis, factors related to lymph nodes metastasis were incorporated into the model. The results were shown in Table 6. Statistical analysis revealed that TMEM16A mRNA expression was an important independent predictive factor of lymph node metastasis in CRC (OR = 16.38, CI: 1.91–140.27, *p* = 0.01). Meanwhile, we found that CD34 and mutant p53 were strong risk factors for lymph node metastasis (CD34: OR = 3.39, CI: 1.13–10.21, *p* = 0.03 and mutant p53: OR = 1.98, CI: 1.05–3.73, *p* =0.04).

**Table 6:** Univariate and multivariate logistic regression model for TMEM16A and LNM.

	**Unitivariate**					
	**TMEM16A mRNA expression**			**TMEM16A protein expression**		
	**OR**	**95% CI**	***p***	**OR**	**95% CI**	***p***
**(A)**						
Gender(Male vs. Female)	0.32	0.11–0.95	0.04	0.54	0.21–1.44	0.22
Age(years; <60 vs. ≥60)	0.81	0.31–2.14	0.68	0.70	0.29–1.71	0.43
Location (colon vs. rectum)	2.31	0.80–6.65	0.12	2.26	0.90–5.69	0.08
Tumor size (T1/T2/T3/T4)	3.34	0.93–12.30	0.07	2.06	0.74–5.74	0.17
Histological type^a^	3.46	0.78–15.42	0.10	3.47	0.96–12.53	0.06
Differentiation (Well/Moderate/Poor)	1.92	0.70–5.27	0.21	2.38	0.98–5.82	0.06
EGFR (Positive/Weakly positive /Negative)	1.36	0.78–2.38	0.28	1.36	0.78–2.39	0.28
CD34 (Positive/Vessel positive/Negative)	2.32	1.10–4.89	0.03	3.16	1.47–6.76	0.01
Ki67^b^	0.66	0.41–1.05	0.08	1.19	0.83–1.70	0.36
mutant p53 protein^c^	1.54	1.05–2.28	0.03	1.69	1.15–2.48	0.01
MLH1^d^	1.61	0.46–5.59	0.46	2.13	0.62–7.39	0.23
MSH6^d^	1.99	0.63–6.29	0.24	3.43	0.78–15.11	0.10
MSH2^d^	1.90	0.40–9.05	0.42			1.00
KRAS (Wild type vs. Mutant type)	1.33	0.53–3.33	0.54	0.85	0.34–2.08	0.71
TMEM16A mRNA (Negative vs. Positive)	3.14	0.95–10.39	0.06			
TMEM16A protein (Negative vs. Positive)				0.48	0.17–1.37	0.17
	**Multivariate**					
	**TMEM16A mRNA expression**			**TMEM16A protein expression**		
	**OR**	**95% CI**	***p***	**OR**	**95% CI**	***p***
**(B)**						
Gender (Male vs. Female)	0.18	0.03–1.07	0.06			
Tumor size (T1/T2/T3/T4)	2.66	0.41–17.39	0.31			
Histological type^a^	11.32	0.66–193.53	0.09	1.50	0.11–20.16	0.76
CD34 (Positive/Vessel positive/Negative)	3.39	1.13–10.21	**0.03**	3.66	1.30–10.30	**0.01**
Ki67^b^	0.67	0.27–1.69	0.39			
mutant p53 protein^c^	1.98	1.05–3.73	**0.04**	2.03	1.08–3.80	**0.03**
TMEM16A mRNA (Negative vs. Positive)	16.38	1.91–140.27	**0.01**			
Location (colon vs. rectum)				4.23	0.65–27.36	0.13
Differentiation(Well/Moderate/Poor)				8.72	1.27–59.82	**0.03**
MSH6^d^				6.26	0.13–298.09	0.35
TMEM16A protein (Negative vs. Positive)				0.43	0.08–2.48	0.35

*LNM, lymph node metastasis; OR, odds ratio; 95% CI, 95% confidence interval; p-value of the logistic regression model*.

a *Adenocarcinoma/Mucinous adenocarcinoma/Signet-ring cell carcinoma*.

b *Positive rate≥90%/Positive rate 80–90%/Positive rate 70–80%/Positive rate 60–70%/Positive rate <60%*.

c *Positive rate≥90%/Positive rate 50–90% /Positive rate <50%*.

d *Positive/Partially positive/Negative*.

*Bold values means P < 0.05, which indicate the statistically significantly difference*.

## Discussion

In this study, we performed a detail analysis of TMEM16A mRNA and protein expression in human CRC tissue samples. Consistent with earlier studies (25–28), TMEM16A mRNA expression was found to be significantly up-regulated in CRC tissues compared with para-cancerous normal tissues, indicating that TMEM16A may participate in the process of carcinogenesis. Furthermore, TMEM16A mRNA expression was detected in 76% CRC cases, whilst TMEM16A protein expression occurred at a lower frequency (26%). There is a weak positive correlation between TMEM16A mRNA expression and protein expression. (Spearman's = 0.337, *p* = 0.019). TMEM16A mRNA expression didn't lead to TMEM16A protein expression in more than half of cases. Although TMEM16A mRNA expression may be responsible for TMEM16A protein expression, it is obviously not the only mechanism of TMEM16A expression in CRC. Similar discrepancys for TMEM16A mRNA and protein expression have been reported in previous studies (19, 25–28). For instance, TMEM16A gene amplification was frequently detected than protein expression in HNSCC samples (25, 27). In contrast, TMEM16A overexpression was more pervasive than gene amplification in human breast cancer and human gastric cancer (27, 29). We will discuss in detail the possible mechanisms leading to this interesting difference later.

The roles of TMEM16A expression in multiple tumor samples has been extensively investigated. Most investigators reported that TMEM16A expression promotes tumor growth and metastasis, and is associated with poor patient prognosis (21, 25–28, 30–34). However, some researchers demonstrated different effects of TMEM16A on these hallmarks. For example, Shiwarski et al. demonstrated that primary tumors exhibit a high level of TMEM16A, whereas metastasis from lymph nodes have a low expression of TMEM16A (28). Wu et al. reported that TMEM16A overexpression is associated with good prognosis in PR-positive or HER2-negative breast cancers patients following Tamoxifen treatment, especially in those patients with the low expression of Ki67 (35). Dixit et al. found that TMEM16A/ANO1 was preferentially overexpressed in HPV negative HNSCC compared with HPV positive HNSCC, and that this overexpression was associated with decreased patient survival (36). Rodrigo et al. observed that there was no correlation between TMEM16A and clinical parameters in HNSSC and patients with TMEM16A-positive oropharyngeal tumors exhibited a significantly improved disease-specific survival, compared to hypopharyngeal, and laryngeal tumors (18). These results indicated the multifaceted role of TMEM16A in various cancers may be cell type-dependent.

The present study is the first conducted to explore the predictive and prognostic value of TMEM16A in patients with CRC by analyzing the correlation between TMEM16A mRNA expression or protein expression and clinical parameters. The results demonstrated that TMEM16A mRNA expression is correlated with tumor TNM stage and lymph node metastasis. The positive rate of TMEM16A mRNA expression in primary colorectal cancer showed a significant increase with lymph node metastasis and late TNM stage. Univariate and multivariate logistic regression analysis suggested that TMEM16A mRNA expression was an important independent predictive factor of lymph node metastasis in CRC. These results showed that TMEM16A mRNA expression may be a promising predictive biomarker just as previously reported (36–38). However, we found that higher levels of TMEM16A mRNA expression mainly occurred in N0 phase and TNM II stage (Supplementary Figure 2), suggesting that TMEM16A mRNA expression level may not be positively correlated with lymph node metastasis and late tumor stage, which was inconsistent with the results by Park et al. (26). Various factors could contribute to these disagreement results, such as differences in the treatment regiments of patient enrollment, particular tumor environment, sample size, and/or detection methods.

We next studied the relationship of TMEM16A expression with clinical prognosis of CRC patients. The results showed that TMEM16A mRNA expression tended to shorter disease-free survival and overall survival for CRC patients, although it is not statistically significant. This could because TMEM16A mRNA expression was closely related to TNM stage and lymph node metastasis, which contribute to clinical prognosis of CRC patients. In addition, other factors such as the patient's age, tumor location, venous invasion, and the treatment after operation, such as radiotherapy, chemotherapy, and targeted therapy, might affect the prognosis of patients. Another possibility is that this may be due to cell type-dependent, which needs to be further confirmed by increasing the sample size and prolonging the observation time of patients.

In order to verify our results, we made further bioinformatics analysis of TMEM16A mRNA expression in TCGA database (http://ualcan.path.uab.edu/). The results were showed in Supplementary Figure 3. The results demonstrated that TMEM16A was upregulated in colon adenocarcinoma (*n* = 286) comparing with normal tissues (*n* = 41), which had statistical significance (*P* = 1.62E−12). There was no significant difference (*p* = 0.36) in overall survival between patients with high TMEM16A mRNA expression (*n* = 70) and those with low TMEM16A mRNA expression (*n* = 209). These results are consistent with our results.

It has been reported that TMEM16A contributed to tumor progression by modulating other factors and their downstream signaling pathways (27, 29, 30, 38–42). However, the mechanisms underlying regulation of tumor tumorigenesis, growth, and metastasis by TMEM16A remained unclear. To explore the potential mechanism, we investigated the correlation between TMEM16A expression and mutation status of KRAS, NRAS, and BRAF, and the protein expression of most commonly used IHC Characteristics including BRAF (V600E), CDX2, EGFR, p53, Ki67, CD34, PMS2, MLH1, MSH6, and MSH2 in clinical CRC sample.

We found that there was a significant positive correlation between MSH2 and TMEM16A mRNA expression. Previous studies have shown that MLH1, MSH2, MSH6, and PMS2 as main proteins of mismatch repair proteins (MMR) are used to repair DNA replication errors. MMR deficient (dMMR) leads to microsatellite instability (MSI), which is an important cause of CRC (43). In the occurrence and development of CRC, 90% of MMR gene mutations are mainly caused by the inactivation of MLH1and MSH2 (44). It seems that MSH2 may be involved in the role of TMEM16A in the occurrence and development of CRC.

Strikingly, our statistical analysis showed that TMEM16A protein expression was positively correlated with KRAS mutation status, and negatively correlated with mutant p53 protein expression. Recent comprehensive genome analyses have identified frequently mutated genes in human CRC, including APC, KRAS, TGFBR2, and Tp53 (45). Among them, KRAS and p53 mutations have been found in ~40 and ~60% of CRC (45, 46). Gene mutations in KRAS and p53 are thought to be essential events for colorectal cancer development. Previous findings suggested that mutated KRAS continuously activates RAS-RAF-MAPK signaling pathway, which leads to uncontrolled cell proliferation and canceration (46, 47). Thus, we infer that TMEM16A protein may promote the occurrence and growth of colorectal cancer by activating mutated KRAS. In addition, it is generally believed that mutant p53 overexpression is related to tumor metastasis, recurrence, and poor prognosis (48, 49). More importantly, our logistic regression analysis further confirmed that mutant p53 protein was an independent predictive factor of lymph node metastasis in CRC samples. Therefore, we speculate that TMEM16A protein might suppress tumor metastasis indirectly by decreasing mutant p53 protein expression. Considering this, our data evoke an intriguing possibility that TMEM16A protein may play a dual role in tumor formation and metastasis by interacting with mutated KRAS and mutant p53 protein in CRC tissues. It should be noted that other proteins might be involved in the regulation of tumor growth and metastasis by TMEM16A, so further studies are required.

Although it has been recently reported that TMEM16A activated EGFR signaling pathway in HNSCC, breast cancer and pancreatic cancer (15, 30, 31, 50, 51). However, in this study, we did not find that TMEM16A was significantly associated with EGFR in human CRC tissues. One of the possible reasons is that TMEM16A regulates cancer cell function via its different protein networks in different cancer cells.

Previous studies demonstrated that TMEM16A expression could be regulated at transcriptional, translational, and post-translational level, and TMEM16A expression is able to modulate different molecules through multiple ways in various cancers (52). Based on our results, we speculated that MSH2 might be involve in regulating of TMEM16A mRNA expression at transcriptional level or act as a regulator in the translation process of TMEM16A, while KRAS and p53 might interact with TMEM16A protein at post-translational level. However, there is no direct evidence to demonstrate how they regulate each other. Here, we found this phenomenon and tried to explain it, but further investigation is needed to elucidate the underlying mechanism. In our study, we observed that with lymph node metastasis and higher TNM stage, the positive rate of TMEM16A mRNA expression increased significantly, but the positive rate of TMEM16A protein did not change significantly. We supposed that the difference might be related to MSH2, KRAS, and p53. In addition, we believe that the coordination of TMEM16A and various factors leads to this result. For example, TMEM16A mRNA expression might be regulated by other factors such as hypermethylation of the TMEM16A promoter, the signal transducer and activator of transcription (STAT) and some soluble factors in the tumor micro-environment at the transcriptional level during lymph node metastasis. TMEM16A expression might be controlled by a number of microRNAs such as miR-9, miR-144, and miR-132 at translational level, as previously reported (13, 26, 52). TMEM16A protein might interact with other molecular targets including ERK1/2, AKT, camodulin kinase II (CaMKII), EGFR, secreted calcium-activated chloride channel regulator 1 (CLCA1), and Coatomer protein complex subunit beta 1 (COPB1) at post-translational level. Due to the heterogeneity of cell subsets expressing different molecules in colorectal cancer, it is necessary to further study the molecular targets involved in the regulation of lymph node metastasis by TMEM16A and the interaction mechanism between TMEM16A and these molecular targets.

## Conclusion

In summary, we newly described the prognostic role of TMEM16A expression and its correlation with clinical pathological parameters. We found that TMEM16A mRNA expression was more frequently detected than TMEM16A overexpression in human colorectal cancer tissue samples. TMEM16A mRNA expression can be used as an independent predictor for lymph node metastasis in CRC. TMEM16A mRNA expression was significantly associated with MSH2 protein. TMEM16A protein expression TMEM16A was positively correlated with KRAS mutation, and negatively correlated with mutant p53 protein. Our finding provides original evidence that TMEM16A mRNA expression may be a potential marker for predicting lymph node metastasis and TMEM16A protein may be a marker between tumor growth and metastasis in CRC.

## Data Availability Statement

The original contributions presented in the study are included in the article/Supplementary Material, further inquiries can be directed to the corresponding authors.

## Ethics Statement

The studies involving human participants were reviewed and approved by the Ethics Committee of the Second Hospital of Jilin University. The patients/participants provided their written informed consent to participate in this study.

## Author Contributions

YS, HL, and QY conducted experimental operations, sample processing, data analysis, and article writing. ZD, FW, HZ, LY, and SC performed the experiments. SH and ZM performed sample pretreatment. YS conceived and designed the experiments. All authors read and approved the final manuscript.

## Conflict of Interest

The authors declare that the research was conducted in the absence of any commercial or financial relationships that could be construed as a potential conflict of interest.
