# Supplementary material for: Expression of TMEM16A in Colorectal Cancer and Its Correlation With Clinical and Pathological Parameters
Source: Front Oncol. 2021 Mar 19;11:652262. doi: 10.3389/fonc.2021.652262 (PMC8017291; doi:10.3389/fonc.2021.652262)
Supplement: Supplementary file 1 [file Data_Sheet_1.docx]

**Supplementary data**


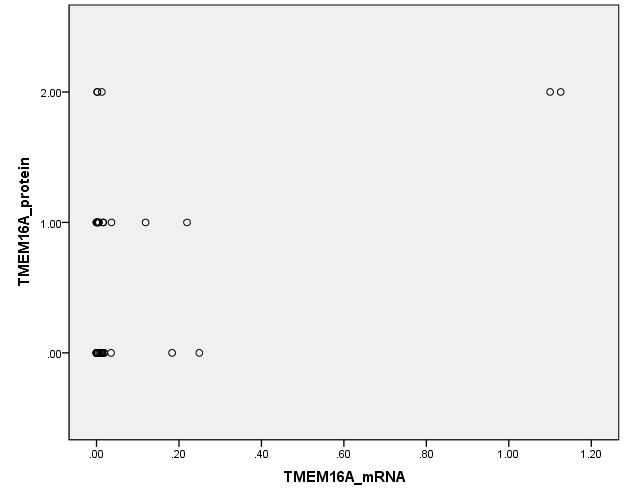


**Figure S1 The correlation of TMEM16A mRNA and protein expression (Spearman’s = 0.337, p = 0.019).**

**
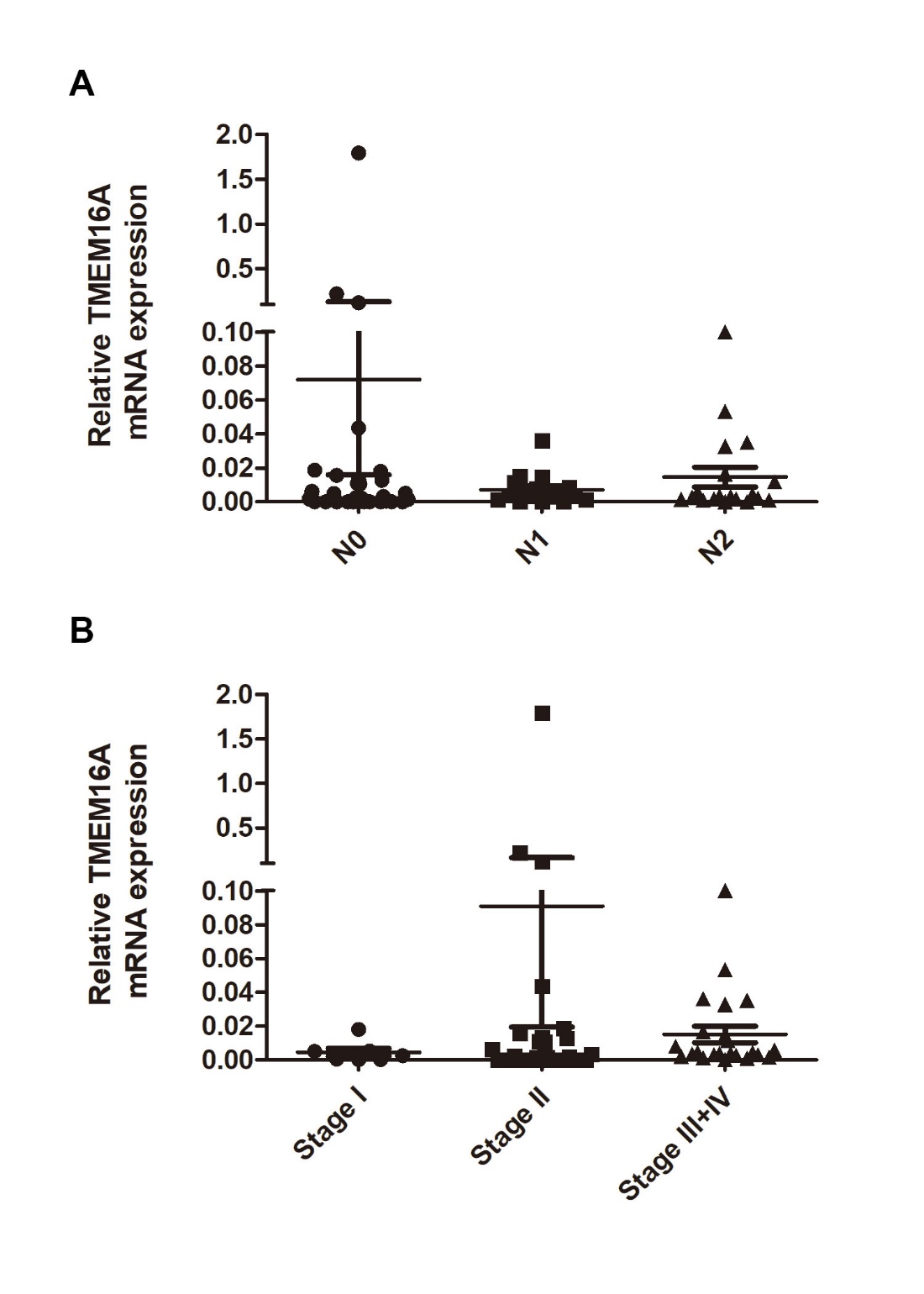
**

**Figure S2 TMEM16A mRNA expression level in different lymph node status and stages. (A) There was no significant correlation between TMEM16A mRNA expression level and lymph node status. (B)** **There was no significant correlation between TMEM16A mRNA expression level and tumor stage.**

**
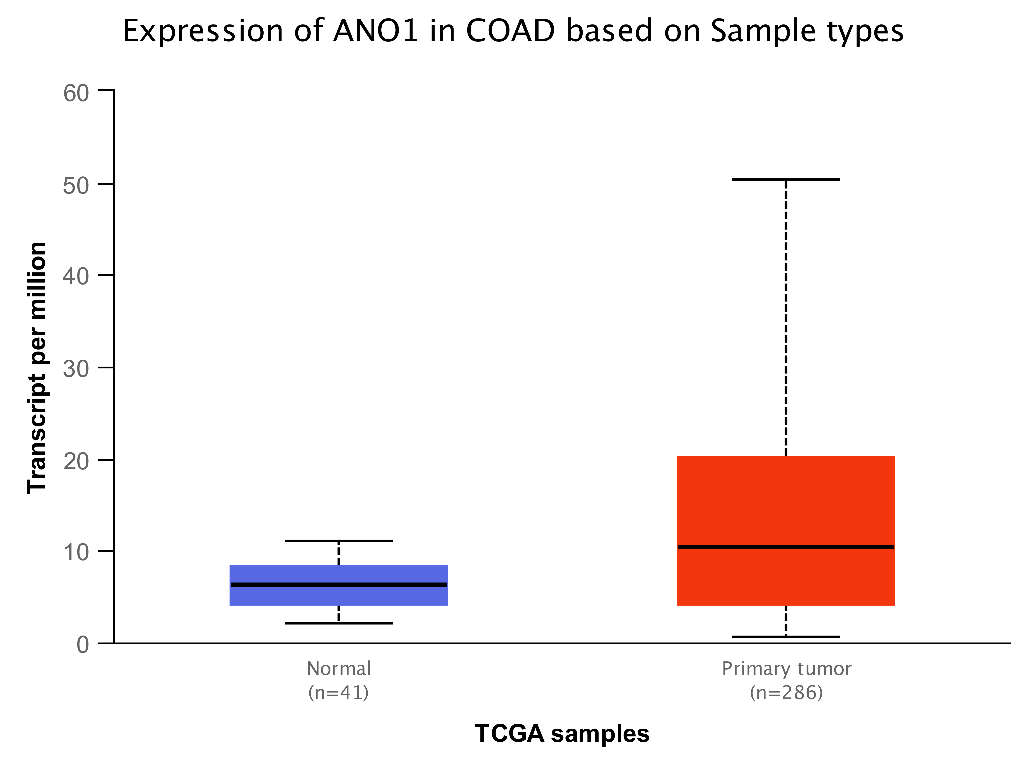
A**


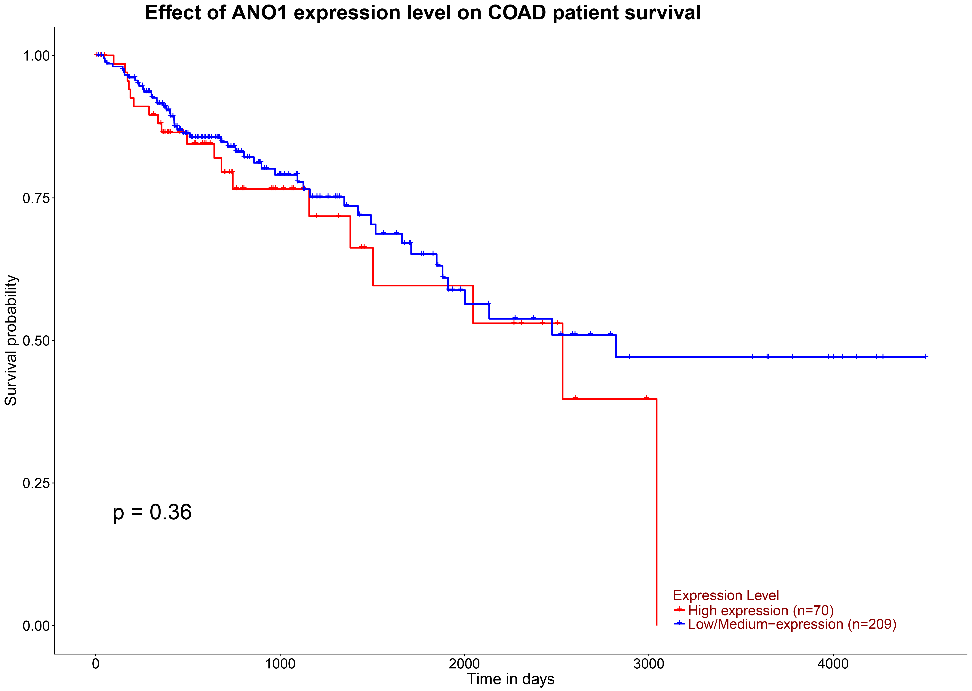
**B**

**Figure S3 Bioinformatics analysis results of TMEM16A mRNA expression in TCGA database (**<http://ualcan.path.uab.edu/>**). COAD: colon adenocarcinoma. (A) There was significant correlation between ANO1/TMEM16A mRNA expression in colon adenocarcinoma (n=286) and normal tissues (n=41). (B) There was no significant correlation between TMEM16A mRNA expression level and** **overall survival of CRC patients.**
